# A Comparative Analysis of Dental Measurements in Physical and Digital Orthodontic Case Study Models

**DOI:** 10.3390/medicina58091230

**Published:** 2022-09-06

**Authors:** Elena-Raluca Baciu, Dana Gabriela Budală, Roxana-Ionela Vasluianu, Costin Iulian Lupu, Alice Murariu, Gabriela Luminița Gelețu, Irina Nicoleta Zetu, Diana Diaconu-Popa, Monica Tatarciuc, Giorgio Nichitean, Ionuț Luchian

**Affiliations:** 1 Department of Implantology, Removable Dentures, Dental Technology, Faculty of Dental Medicine, University of Medicine and Pharmacy “Grigore T. Popa”, 700115 Iasi, Romania; 2 Department of Surgery, Faculty of Dental Medicine, University of Medicine and Pharmacy “Grigore T. Popa”, 700115 Iasi, Romania; 3 Faculty of Dental Medicine, University of Medicine and Pharmacy “Grigore T. Popa”, 700115 Iasi, Romania; 4 Department of Periodontology, Faculty of Dental Medicine, University of Medicine and Pharmacy “Grigore T. Popa”, 700115 Iasi, Romania

**Keywords:** dental study model, additive manufacturing, direct light processing, arch measurements

## Abstract

*Background and Objectives:* Study models are essential tools used in the dental teaching process. The aim of the present study was to compare the values obtained by manual and digital orthodontic measurements on physical and digital case study models. *Materials and Methods:* The physical experimental models were obtained by traditional pouring (improved stone-type IV gypsum products) and by additive manufacturing (resins). The digital experimental models were created by scanning the physical ones, using a white light-emitting diode (LED) source and an L-shaped dental scanner—Swing DOF (DOF, Seoul, Korea). The physical study models were first measured using a digital caliper, and then, they were scanned and evaluated using the DentalCad 3.0 Galway software (exocad GmbH, Darmstadt, Germany). The Pont, Linder–Harth, and Bolton indices, which are used in orthodontics for training students, were derived using the available data. *Results:* When comparing the linear measurement mean ranks taken on physical study models to those of digital models, no statistically significant differences (*p* > 0.05) were found. A similar result was also shown when the dentoalveolar growth indicators were analyzed. *Conclusions:* It can be concluded that dental study models made by direct light processing (DLP) and pouring type IV class gypsum are both acceptable for orthodontic teaching purposes.

## 1. Introduction

Dental models are an indispensable diagnostic and legal tool for all dental disciplines regarding the processes for training future dentists. They may also be used as a documentary tool, working well as a duplicate model. Furthermore, plaster models are valued by the academic community for their use in evaluating patient progress and documenting research [1,2].

Traditionally, dental models are made in the laboratory using gypsum products with different levels of hardness, depending on the model’s purpose. These are obtained from dental arch impressions—which are recorded using elastic materials or intraoral scanners—producing positive images of a patient’s teeth and the surrounding tissue, which must be reproduced as accurately as possible. Intraoral scanners are becoming more and more common, but little is known about their accuracy for full-arch scans, despite their increasing use in daily life [3,4]. The accuracy of a scan is affected by intraoral conditions, such as the optical digitalization unit’s restricted area, possible fogging of the digitalization unit, the patient’s and dentist’s movements, intraoral light, the presence of humidity (saliva or blood), the soft tissue, or the optical scanning equipment used (scanning wands) [5].

Traditional stone dental models have notable advantages, including their affordability, simplicity of use, accuracy in details impression reproduction, compatibility with impression materials, dimensional stability, and great mechanical properties. The disadvantages of using them include the need for additional storage space and the risk of fracture and deterioration [6].

In contrast, digital models have a number of advantages, such as low cost, less time consumed, the ability to share online images with other practitioners and patients, the fact that they are durable and not prone to degradation, lower laboratory and chairside expenses, computerized storage, enhanced patient instruction, and better professional productivity and efficiency [7,8,9]. In cases of periodontal patients, digital dental models are recommended, as they reduce the trauma caused by the impression procedure [10].

Despite these benefits, digital models are not yet routinely used in daily practice because of some disadvantages in their application, including data loss in cases of degradation in electronic storage, dependence on third parties, time-consuming software support, the need to learn the operating system, and high equipment costs.

The additive rapid prototyping/three-dimensional (3D) printing methods most often used in dentistry are stereolithography (SLA), digital light processing (DLP), selective laser sintering (SLS), selective laser melting (SLM), electron beam processing (EBM), PolyJet photopolymer printing, and fused deposition modeling (FDM) [11,12,13].

Both DLP and SLA printing techniques employ similar printing principles (layer-by-layer solidification of a light-polymerizable liquid polymer under laser illumination) but require different devices [14]. To build extremely accurate models with fine-grained geometries, SLA makes use of a moving ultraviolet (UV) laser beam, whereas DLP makes use of fixed UV light from a projector [15,16]. The polymerization of each multilayer resin deposit occurs far more quickly (in a matter of seconds) than with SLA, making DLP a preferable process for dental laboratories with an industrial character. It also has a lower cost than SLA by saving material (photopolymers and ceramic-filled resins) [17]. The digital light-processing indications are as follows: dental models, cast coping, resin patterns, wax pattern splints, temporary restorations, surgical guides, aligners, retainers, and castable crowns and bridges [18,19,20,21].

The accuracy of printed models may vary greatly due to the materials (aging process), equipment, and procedures used in the technical fabrication processes [22].

The degree of dental arch development may be assessed by looking at the size and space of the teeth in relation to one another [23]. Many variables, both hereditary and environmental, can lead to dental anomalies and occlusal problems [24]. Malocclusions can be induced by volume anomalies, which are one of the contributing factors. Incidence of different dental defects are explored in several studies; however, few focused on malocclusions in relation to the teeth [25].

During the educational orthodontic evaluation of the study models, the Linder–Harth and Pont indices and the Bolton analysis are frequently used. The Linder–Harth approach, which was developed from Pont’s index, is useful for estimating the width of the dental arch. These methods employ both measured and calculated values based on formulas [26,27]. When establishing the difference between these values, it is possible to determine whether or not there is a dental arch abnormally present in transversal planes. However, the transverse sizes of dental arches are not governed by the size of the teeth but by the gnathic type of the arch [27].

Bolton’s analysis was developed in 1958 and assesses whether there is a volume difference between the maxillary and mandibular permanent teeth. Bolton [28] also discovered a relationship between the total mesiodistal widths of the teeth and suggested that an anterior ratio value of 77.2% and overall ratio value of 91.3% are needed for ideal occlusion. The frontal ratio is the only one that can be determined in cases in which there are edentulous spaces in the lateral area.

A difference of more than 1.5 mm in the size of mandibular and maxillary teeth is clinically important, with involvement in the future treatment plan [29].

The purpose of the present study is to compare the values obtained by traditional and digital orthodontic measurements on physical and digital case study models.

In order to achieve the proposed objective, we formulated two null hypotheses:

– the linear measurement values are not influenced by the method, the material, or the obtainment technique used in the case study models;
– the lack of space is undisturbed by the obtained values using various measurement techniques and dental study models.

## 2. Materials and Methods

### 2.1. Patient Selection and Impression Recording

**Figure 1:**
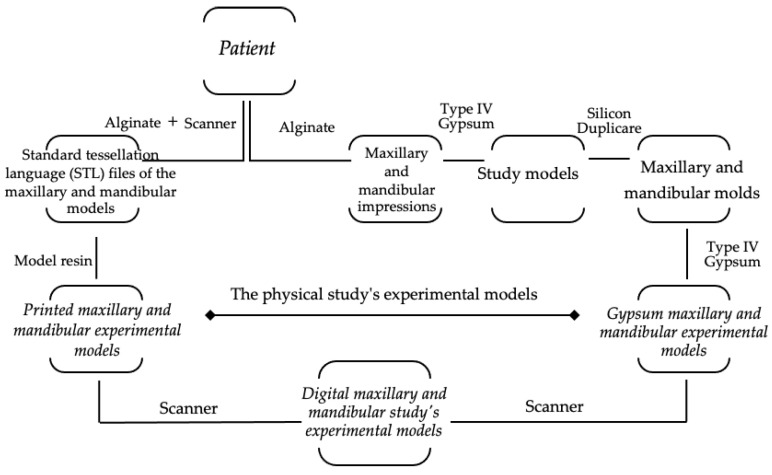
The study protocol’s design: from patient to digital case study models.

The study protocol (Figure 1) was approved by the Ethics Committee of “Grigore T. Popa” University of Medicine and Pharmacy of Iasi (No. 196/03.06.2022), and the included participants consented to the procedures.

To perform the experimental models, alginate maxillary and mandibular impressions were taken from patients.

Patients had to meet the following criteria in order to be included in the study: over 18 years of age; cooperative; no general diseases; no previous experience of anaphylactic reactions; completely erupted permanent dentition from the first molar; no interproximal caries or fillings, prosthetic crowns, or bridges; no teeth anomalies, edentation, orthopedic, or orthodontic treatments in their history; and diagnosed with Class I malocclusion.

The impressions were registered at the Faculty of Dentistry, Iasi, Romania. To achieve this stage, medium-sized plastic–steel impression trays (Guangzhou Aurora Health Products Company, Hunan, China), perforated for a better retention of the impression material, together with Orthoprint (Zhermack SpA, Badia Polesine, Italy) alginate material, were used. After the appropriate amount of powder with water was measured out and prepared in accordance with the manufacturer’s recommendations, the final impressions were transported to the dental laboratory, in a 100% humidity medium, within 30 min, and then poured [30].

### 2.2. The Methods of Producing the Case Study Models

#### 2.2.1. The Physical Models

**Figure 2:**
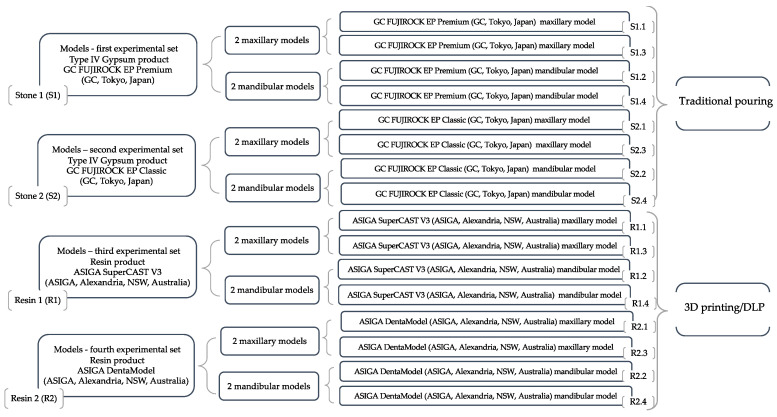
Experimental case study models.

Traditional pouring and additive manufacturing/digital light processing were used to create 4 sets of experimental models. Each set (using same material and method) included 4 study models—2 maxillary and 2 mandibular; therefore, a total of 16 models were obtained (Figure 2).

##### Dental Stone Models

Silicon Duplicate Elite Double 22 (Zhermack SpA, Badia Polesine, Italy) was used to duplicate the models. When using Type IV gypsum powder, the manufacturer’s recommended dosage of water was followed. For 30 s, the gypsum paste was molded under a vacuum to produce a homogenous paste with a semi-fluid consistency, free of air inclusions, after it was spatulated. The gypsum material was gradually poured in the mold placed on the vibration table. After 60 min, the models were removed from the molds and stored for 48 h at room temperature [31]. All models were poured by the same dental technician.

##### Three-Dimensionally Printed Models

The scanned images (Swing DOF Scanner—DOF, Seoul, Korea) of the recorded impressions were automatically converted to standard tessellation language (STL) format by the dedicated scanner software. The files were then imported using the Asiga Composer software, version 1.2 (ASIGA, Alexandria, NSW, Australia) to be manufactured on a digital light processing printer, 3D MAX UV Asiga (ASIGA, Alexandria, NSW, Australia).

The following settings were used:

– Support scripts: contact with the model—0.5 mm; height leveling—2 mm; support spacing—2 mm; material strength—40×; and torsion tolerance—0.
– Thickness layer—0.05 mm.
– “Fast print” mode with separation detection and anti-aliasing.

**Figure 3:**
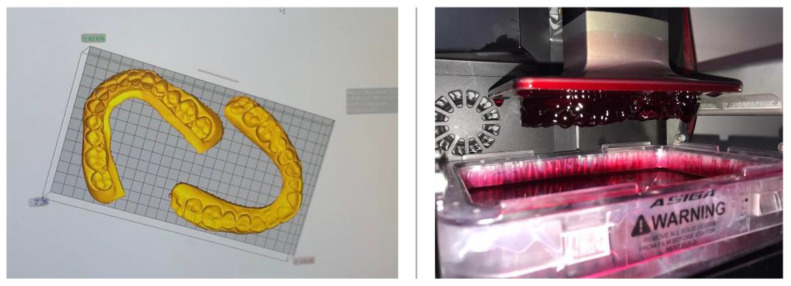
The 3D-printed models: from STL files to printed models.

At each printing cycle, two models in a series were printed in a horizontal position (Figure 3). Each cycle lasted for approximately one hour. The printed models were stored for 24 h at room temperature [22].

#### 2.2.2. The Digital Models

The digital models were created by scanning the physical ones, using a white light LED source and an L-shaped dental scanner—swing DOF (DOF, Seoul, Korea). The data were saved in an STL-format file.

### 2.3. Dental Measurement

We evaluated the reproducibility of dental arch characteristics, such as mesiodistal widths of incisors, canines, premolars, and first molars, as well as interpremolar and molar widths, using manual and digital linear measurements as follows:

– The upper arch interpremolar width was measured between central grooves on the occlusal surface of the first premolars.
– The superior intermolar distance was measured between mesial pits on the occlusal surface of first molars.
– The distance between the contact points of the lower premolars was assessed for the lower premolar diameter.
– The distance between the tips of the distobuccal cusps of the first lower molar was used as the point of measurement for the lower molar diameter [26,27,28].

#### 2.3.1. Traditional Dental Measurement

The measurements were taken with a portable digital caliper Gedore No. 711 (GEDORE Austria GmbH, Österreich, Austria) with 0.01 mm accuracy, which was previously calibrated. Each measurement was performed twice, at one-day intervals, by the same operator. The operator was instructed to measure a maximum of 8 dental study models in a single day so that fatigue related to errors may be reduced. The procedure was repeated in order to include all of the models, and a Microsoft Excel spreadsheet was used to record the results of the measurements that were taken in millimeters. A total of 448 manual measurements were performed.

#### 2.3.2. Modern Dental Measurement

**Figure 4:**
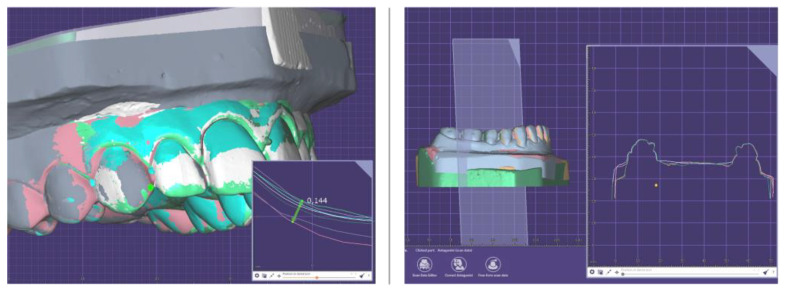
DentalCAD 3.0 Galway (exocad GmbH, Darmstadt, Germany) measurements on the digital study models.

The digital measurements made on the scanned experimental dental models followed the same guidelines. The digital models were analyzed using DentalCAD 3.0 Galway (exocad GmbH, Darmstadt, Germany). The three-dimensional images were rotated and enlarged on screen to facilitate measurements (Figure 4).

A total of 14 measurements were made on each dental digital model by the same operator.

### 2.4. Orthodontic Model Analysis

Using these measured values for the orthodontic model analysis [26,27,28], the Pont and Linder–Harth indices, Bolton’s anterior, and the overall ratio were calculated (Table 1).

### 2.5. Statistical Analysis

Statistical analysis was performed using SPSS, version 20 (SPSS Inc., Chicago, IL, USA). The obtained data were subjected to multiple Mann–Whitney U tests for pairwise comparisons among groups represented by manual and digital measurements on physical and digital models. The statistical analysis was conducted at a significance level of *p* < 0.05.

## 3. Results

### 3.1. Evaluation of the First Hypothesis

The following data were compared in order to test the first study hypothesis:

– The average values obtained by manual measurements of the mesiodistal widths of the incisors, canines, premolars, and first permanent molars, as well as the interpremolar and molar widths at the level of the traditionally models (type IV gypsum) versus 3D-printed models (resins).
– The average values acquired by digital measurements of the mesiodistal widths of the incisors, canines, premolars, and first permanent molars, as well as the interpremolar and molar widths at the level of scanned models: type IV gypsum digital model versus resin (3D printing) digital model.
– The average values produced by manual measures as opposed to digital measurements of the mesiodistal widths of the incisors, canines, premolars, and first permanent molars, as well as the interpremolar and molar widths at the level of the traditional models (type IV gypsum).
– The average values obtained by manual measurements, as opposed to digital measurements, of the mesiodistal widths of the incisors, canines, premolars, and first permanent molars, as well as of the interpremolar and molar widths at the level of the additive processing models (resins).

The findings from the statistical analysis for the maxillary and mandibular arches are shown in Table 2 and Table 3.

Even if there were differences in the mean ranks of the obtained results (with greater values recorded in digital measurements), they were not statistically significant (*p* > 0.05).

### 3.2. Evaluation of the Second Hypothesis

In order to evaluate the second hypothesis, the following values were compared:

– The values obtained by manual and digital measurements of the Pont index, the Linder–Harth index, and Bolton’s analysis on traditionally poured versus 3D-printed models.
– The values obtained by manual versus digital measurements of the Pont index, the Linder–Harth index, and Bolton’s analysis on physical and digital models.

**Figure 5:**
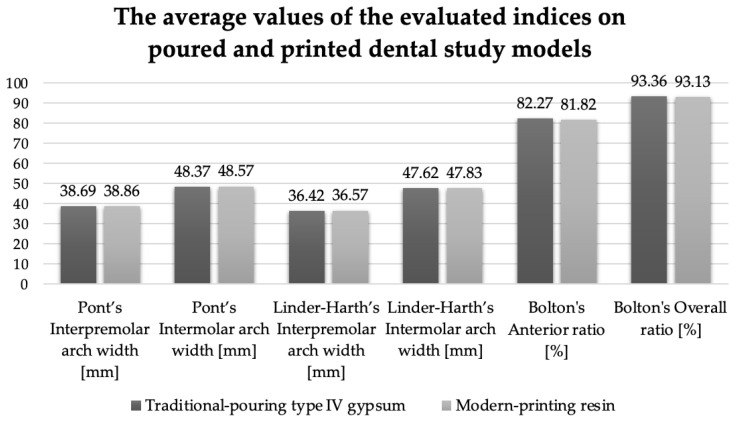
The average values of the evaluated indices on the experimental case study models.

**Figure 6:**
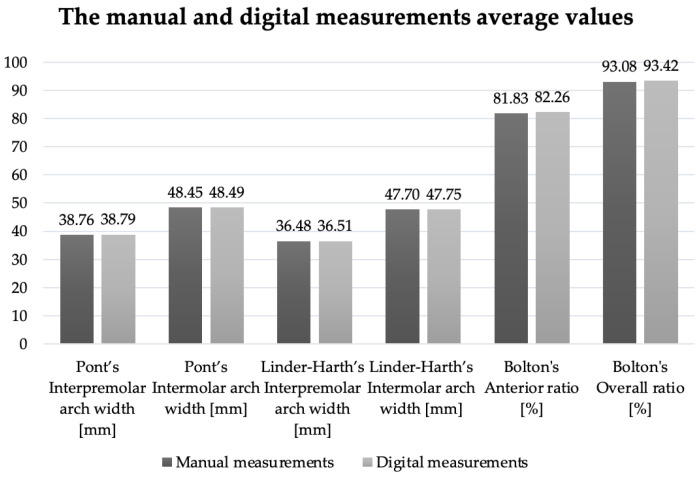
The average values of the evaluated indices by manual and digital measurements.

A similar result for excessive mesiodistal mandibular teeth (Bolton’s overall ratio > 91.3%) was noticed from a comparative examination of the average values obtained in the case of the investigated indices (Figure 5 and Figure 6).

Table 4 shows statistically significant values for the measured indices: Pont interpremolar and intermolar arch widths (*p* < 0.05) and Linder–Harth interpremolar and intermolar arch widths (*p* < 0.05) at the level of printed models versus traditional ones. When the difference between the calculated and the measured values was evaluated, a statistically insignificant result (*p* = 0.83—Pont’s and Linder–Harth’s interpremolar arch widths; *p* = 0.59—Pont’s and Linder–Harth’s intermolar arch widths) was obtained.

In the case of the overall ratio (Bolton’s analysis, *p* < 0.05), a statistical significance between the mean ranks of manual versus digital measurements (Table 5) was established. However, the difference between the average values was 0.34 mm, which is considered to have no clinical significance.

## 4. Discussion

Numerous authors highlighted dental digital models as beneficial. Some of these advantages include simpler data transmission, reduced treatment planning, and shorter diagnostic time when compared with traditional model setups and reconstruction [32,33,34]. However, when considering the usefulness of digital models, the following question arises: are they reliable?

Two previous systematic reviews, by Fleming et al. [35] and Luu et al. [36], respectively, compared the validity of digital model measurements with those from plaster models. According to the authors’ findings, the digital model evaluations were correct.

The study results show that there were no statistically significant differences in the mean rank of the obtained linear measurement values on the physical and digital case study models, which means that the first null hypothesis was verified.

Similar findings were obtained by Sousa et al. [37], who evaluated the reliability of measurements made on 3D digital models obtained by scanning plaster models with laboratory scanners. The authors emphasized the increased ability to enlarge and rotate the pictures of the digital model image, as well as the software’s simplicity of use in detecting landmarks.

Abizadeh et al. [6] found a statistically significant difference between model analysis on plaster models and digital models created by model scanning. Measurements of plaster models were more accurate than measurements of digital models due to the fact that the digital model scans were not a true 1:1 replica of the plaster ones.

The current investigation included study models obtained by full dental arch impressions with alginate material. The results indicate that printed and traditional models both properly reproduce dental arch details. In contrast, a recent study conducted by Sayed et al. [38] concluded that stone casts generated using polyvinyl siloxane and alginate impression and pouring type IV die stone have a higher linear dimensional accuracy than 3D-printed casts.

According to Nestler et al. [39], both extrusion-based and photopolymerization-based printers were precise, although Asiga MAX UV (ASIGA, Alexandria, NSW, Australia) had the highest accuracy. In contrast, Sayed et al. [38] found that the greatest number of distortions above 0.5% were produced by the digital model with full-arch-prepared abutment teeth obtained using the same printer.

Choi et al. [40] and Jin et al. [41] found no statistically significant differences between measurements taken from the physical plaster and printed models using the stereolithography method.

In a systematic review of the literature, Etemad-Shahidi et al. [42] evaluated the accuracy of full-arch dental models manufactured using different 3D-printing technologies and concluded that other factors, such as the layer thickness, base design, postprocessing, and storage can equally influence the accuracy of the resultant 3D-printed models.

It is well documented in the literature that tooth size differences (TSD) play an important role in orthodontic finalization, particularly in the front area. Knowing about TSD and other variables provides the practitioner an advantage when making a final treatment selection to achieve great results.

The existing studies on TSD used traditional measuring compasses or digital calipers to estimate mesiodistal tooth widths using plaster or digital models [43,44].

Furthermore, it is demonstrated that measurements taken from 3D digital models are a viable alternative to those taken from physical models, since storing records is faster, more reliable, and easier to complete. Accuracy is measured using digital calipers, which are widely regarded to be the gold standard [44,45,46,47,48,49].

According to the “clinically acceptable” term [48,50,51,52], the results of the present study reveal that the values had differences of less than 0.5 mm between traditional and 3D-printed models, as well as between manual and digital measurement methods. On the other hand, for prosthodontic applications, the accuracy requirements for dental models are often greater, and a measurement discrepancy of less than 0.2 mm was shown to be clinically acceptable in [53].

Despite minor differences in the measurements of mesiodistal tooth width and arch length on digital models, Leifert et al. [54] found that digital models were clinically acceptable and repeatable when compared to traditional models.

Wan Hassan et al. [50] questioned the accuracy of dental measurements in various degrees of crowded dentitions when measuring stone casts and reconstructed rapid prototyping models.

The findings of the current study show greater mesiodistal teeth width values recorded in digital rather than manual measurements. Similar results were also obtained by Cuperus et al.’s [55] research using an intraoral scanner to create the digital models.

The difference between manual and digital recordings, according to Naidu et al. [48], is explained by the absence of a physical barrier when placing measurement points on virtual models; the difficulty in scanning the contact points, which results in small amounts of missing data that must be interpolated by a computer algorithm; and the operator’s training and proficiency, which can cause minor variations in contact point locations between the stone and digital models.

Even if—as in the case of Pont and Linder–Harth interpremolar and intermolar arch widths (*p* < 0.05) and Bolton’s overall ratio (*p* < 0.05)—a statistically significant difference between the manual and digital measurements was observed, the discrepancies were deemed to have no clinical implications. In this context, the second null hypothesis must also be accepted.

The reasons for the significant differences between physical and digital study models could be a highlighted correction of tooth position, the increased accuracy of the virtual setup compared with the manual one, and the superimposition of moving objects that may affect the geometry of digital models [48,56].

Similar to other in vitro studies, this research had several limitations. One limitation was that only one laboratory scanner, one type of 3D printer, and one software for digital measurements were employed. Another limitation was the difficulty of measuring tooth widths with a digital caliper on physical mandibular models due to access and the difficulty of resting at the exact mesial and distal landmarks in crowded areas.

Digital technology limitations were represented by the scanning procedure (the accuracy of physical models may be affected when imaging powder is applied to them before scanning), the “shape assumption” problem, which occurs when the software uses a computer algorithm to fill the interproximal inaccurate or uncaptured data, and the process of printing, which can produce its own errors [57,58].

Additional research is needed to evaluate the accuracy of dental case study models obtained using various scanners (intraoral and laboratory), printers, and production parameters, with measurements made using dedicated applications.

## 5. Conclusions

Within the limitations of the current study, it can be concluded that the precision of digital measurements of teeth widths, using DentalCAD 3.0 Galway (exocad GmbH, Darmstadt, Germany) on digital models, was comparable to direct measurements with a portable digital caliper Gedore No. 711 (GEDORE Austria GmbH, Österreich, Austria) on physical dental models.

Digital measurements of mesiodistal teeth width showed higher values compared with manual ones; therefore, the difference between the average values recorded had no clinical significance.

For orthodontic teaching purposes, dental study models manufactured by direct light processing (DLP) and traditional pouring are both acceptable.

## Figures and Tables

**Table 1:** The formulas used to calculate the development of the arches.

Methods	Equations
Pont index	Sum of incisors (SI) = sum of mesio-distal width of the maxillary incisors
	Interpremolar arch widths $=\frac{\mathrm{sum}\;\mathrm{of}\;\mathrm{the}\;\mathrm{widths}\;\mathrm{of}\;\mathrm{the}\;\mathrm{maxillary}\;\mathrm{incisors}\times 100}{80}$
	Intermolar arch widths $=\frac{\mathrm{sum}\;\mathrm{of}\;\mathrm{the}\;\mathrm{widths}\;\mathrm{of}\;\mathrm{the}\;\mathrm{maxillary}\;\mathrm{incisors}\times 100}{64}$
Linder–Harth index	Interpremolar arch widths $=\frac{\mathrm{sum}\;\mathrm{of}\;\mathrm{the}\;\mathrm{widths}\;\mathrm{of}\;\mathrm{the}\;\mathrm{maxillary}\;\mathrm{incisors}\times 100}{85}$
	Intermolar arch widths $=\frac{\mathrm{sum}\;\mathrm{of}\;\mathrm{the}\;\mathrm{widths}\;\mathrm{of}\;\mathrm{the}\;\mathrm{maxillary}\;\mathrm{incisors}\times 100}{65}$
Bolton’s analysis	Anterior ratio $=\frac{\mathrm{sum}\;\mathrm{of}\;\mathrm{the}\;\mathrm{widths}\;\mathrm{of}\;\mathrm{the}\;6\;\mathrm{mandibular}\;\mathrm{anterior}\;\mathrm{teeth}}{\mathrm{sum}\;\mathrm{of}\;\mathrm{the}\;\mathrm{widths}\;\mathrm{of}\;\mathrm{the}\;6\;\mathrm{maxillary}\;\mathrm{anterior}\;\mathrm{teeth}}\times 100$
	Overall ratio $=\frac{\mathrm{sum}\;\mathrm{of}\;\mathrm{the}\;\mathrm{widths}\;\mathrm{of}\;\mathrm{the}\;12\;\mathrm{mandibular}\;\mathrm{teeth}}{\mathrm{sum}\;\mathrm{of}\;\mathrm{the}\;\mathrm{widths}\;\mathrm{of}\;\mathrm{the}\;12\;\mathrm{maxillary}\;\mathrm{teeth}}\times 100$

**Table 2:** Maxillary pairwise comparison of the 14 studied diameters using the Mann–Whitney U test.

Pairwise Comparison		Mean Rank	*p*-Value ^a^
Type IV gypsum versus resin DigitalCalliper	S1.1 + S1.3 + S2.1 + S2.3 DigitalCalliper	28.45	0.980
	R1.1 + R1.3 + R2.1 + R2.3 DigitalCalliper	28.55	
Type IV gypsum versus resin Exocad	S1.1 + S1.3 + S2.1 + S2.3 Exocad	28.75	0.909
	R1.1 + R1.3 + R2.1 + R2.3 Exocad	28.25	
DigitalCalliper versus Exocad Type IV gypsum products	S1.1 + S1.3 + S2.1 + S2.3 DigitalCalliper	28.50	1.000
	S1.1 + S1.3 + S2.1 + S2.3 Exocad	28.50	
DigitalCalliper versus exocad Resins	R1.1 + R1.3 + R2.1 + R2.3 DigitalCalliper	28.70	0.928
	R1.1 + R1.3 + R2.1 + R2.3 Exocad	28.30	

^a^ The Mann–Whitney U test was used. The significance level was set at 0.05; S1.1—maxillary stone (GC FUJIROCK EP Premium—GC, Tokyo, Japan) model, no.1; S1.3—maxillary stone (GC FUJIROCK EP Premium—GC, Tokyo, Japan) model, no.3; S2.1—maxillary stone (GC FUJIROCK EP Classic—GC, Tokyo, Japan) model, no.1; S2.3—maxillary stone (GC FUJIROCK EP Classic—GC, Tokyo, Japan) model, no.3; R1.1—maxillary resin (ASIGA SuperCAST V3—ASIGA, Alexandria, NSW, Australia) model no.1; R1.3—maxillary resin (ASIGA SuperCAST V3—ASIGA, Alexandria, NSW, Australia) model no.3; R2.1—maxillary resin (ASIGA DentaModel—ASIGA, Alexandria, NSW, Australia) model no.1; and R2.3—maxillary resin (ASIGA DentaModel—ASIGA, Alexandria, NSW, Australia) model no.3.

**Table 3:** Mandibular pairwise comparison of the 14 studied diameters using the Mann–Whitney U test.

Pairwise Comparison		Mean Rank	*p*-Value ^a^
Type IV gypsum versus resin DigitalCalliper	S1.2 + S1.4 + S2.2 + S2.4 DigitalCalliper	28.71	0.922
	R1.2 + R1.4 + R2.2 + R2.4 DigitalCalliper	28.29	
Type IV gypsum versus resin Exocad	S1.2 + S1.4 + S2.2 + S2.4 Exocad	29.09	0.787
	R1.2 + R1.4 + R2.2 + R2.4 Exocad	27.91	
DigitalCalliper versus Exocad Type IV gypsum products	S1.2 + S1.4 + S2.2 + S2.4 DigitalCalliper	27.95	0.799
	S1.2 + S1.4 + S2.2 + S2.4 Exocad	29.05	
DigitalCalliper versus Exocad Resins	R1.2 + R1.4 + R2.2 + R2.4 DigitalCalliper	28.23	0.902
	R1.2 + R1.4 + R2.2 + R2.4 Exocad	28.77	

^a^ The Mann–Whitney U test was used. The significance level was set at 0.05; S1.2—mandibular stone (GC FUJIROCK EP Premium—GC, Tokyo, Japan) model no.2; S1.4—mandibular stone (GC FUJIROCK EP Premium—GC, Tokyo, Japan) model no.4; S2.2—mandibular stone (GC FUJIROCK EP Classic—GC, Tokyo, Japan) model no.2; S2.4—mandibular stone (GC FUJIROCK EP Classic—GC, Tokyo, Japan) model no.4; R1.2—mandibular resin (ASIGA SuperCAST V3—ASIGA, Alexandria, NSW, Australia) model no.2; R1.4—mandibular resin (ASIGA SuperCAST V3—ASIGA, Alexandria, NSW, Australia) model no.4; R2.2—mandibular resin (ASIGA DentaModel—ASIGA, Alexandria, NSW, Australia) model no.2; and R2.4—mandibular resin (ASIGA DentaModel—ASIGA, Alexandria, NSW, Australia) model no.4.

**Table 4:** Traditional pouring versus 3D printing comparison of the analyzed indices using the Mann–Whitney U test.

Orthodontic Analysis		Traditional Pouring Versus 3D Printing	Mean Rank	*p*-Value ^a^
Pont index	Interpremolar arch widths	Traditional pouring	2.50	0.020 *
		3D printing	6.50	
	Intermolar arch widths	Traditional pouring	2.50	0.020 *
		3D printing	6.50	
	The difference between the calculated and the measured interpremolar arch widths values	Traditional pouring	6.00	0.083
		3D printing	3.00	
	The difference between the calculated and the measured intermolar arch widths values	Traditional pouring	6.00	0.059
		3D printing	3.00	
Linder–Harth index	Interpremolar arch widths	Traditional pouring	2.50	0.021 *
		3D printing	6.50	
	Intermolar arch widths	Traditional pouring	2.50	0.020 *
		3D printing	6.50	
	The difference between the calculated and the measured interpremolar arch widths values	Traditional pouring	6.00	0.083
		3D printing	3.00	
	The difference between the calculated and the measured intermolar arch widths values	Traditional pouring	6.13	0.059
		3D printing	2.88	
Bolton’s analysis	Anterior ratio	Traditional pouring	5.75	0.149
		3D printing	3.25	
	Overall ratio	Traditional pouring	5.63	0.189
		3D printing	3.38	

^a^ The Mann–Whitney U test was used. * The significance level was set at 0.05.

**Table 5:** Manual versus digital measurement comparison of the analyzed indices using the Mann–Whitney U test.

Orthodontic Analysis		Manual Versus Digital Measurements	Mean Rank	*p*-Value ^a^
Pont index	Interpremolar arch widths	Manual measurements	4.50	1.000
		Digital measurements	4.50	
	Intermolar arch widths	Manual measurements	4.50	1.000
		Digital measurements	4.50	
	The difference between the calculated and the measured interpremolar arch widths values	Manual measurements	5.75	0.149
		Digital measurements	3.25	
	The difference between the calculated and the measured intermolar arch widths values	Manual measurements	4.63	0.885
		Digital measurements	4.38	
Linder–Harth index	Interpremolar arch widths	Manual measurements	4.50	0.885
		Digital measurements	4.50	
	Intermolar arch widths	Manual measurements	4.50	1.000
		Digital measurements	4.50	
	The difference between the calculated and the measured interpremolar arch widths values	Manual measurements	5.75	0.149
		Digital measurements	3.25	
	The difference between the calculated and the measured intermolar arch widths values	Manual measurements	4.63	0.885
		Digital measurements	4.38	
Bolton’s analysis	Anterior ratio	Manual measurements	3.00	0.083
		Digital measurements	6.00	
	Overall ratio	Manual measurements	2.63	0.028 *
		Digital measurements	6.38	

^a^ The Mann–Whitney U test was used. * The significance level was set at 0.05.

## Data Availability

The data that support the findings of this study are available on request from the corresponding author.
